# Anthelmintic and Antibiotic Therapy Resolves Intestinal Inflammatory Infiltration in Experimental Trichuriasis

**DOI:** 10.1021/acsinfecdis.5c00596

**Published:** 2025-09-30

**Authors:** Tathiane de Oliveira Alves Costa, Dayane Alvarinho de Oliveira, Alessandra Campos da Silva, Eduardo José Lopes-Torres

**Affiliations:** † Laboratório de Helmintologia Romero Lascasas Porto, Departamento de Microbiologia, Imunologia e Parasitologia, Faculdade de Ciências Médicas, 28130 Universidade do Estado do Rio de Janeiro, Rio de Janeiro, RJ 20550-900, Brazil; ‡ Laboratório Multiusuário de Parasitologia Prof. José Roberto Machado e Silva, Departamento de Microbiologia, Imunologia e Parasitologia, Faculdade de Ciências Médicas, 28130 Universidade do Estado do Rio de Janeiro, Rio de Janeiro, RJ 20550-900, Brazil

**Keywords:** helminth, *Trichuris muris*, antibiotic, anthelmintic, bacterial translocation

## Abstract

Despite significant advances in human health, soil-transmitted helminths (STH) continue to pose a major public health challenge, particularly in impoverished regions. Albendazole has been used to treat STH for over 40 years and remains widely utilized in mass drug administration programs. However, it is estimated that over 1.5 billion people are still infected globally, with Brazil reporting a prevalence of 5.41% for human trichuriasis. The nematode *Trichuris muris* is widely used in murine models to study trichuriasis due to its impact on the epithelial mucosa, including tissue damage, dysbiosis, bacterial translocation, inflammatory infiltrate, and intestinal layer hypertrophy. These effects contribute to the more severe consequence of high parasite load infections, such as rectal prolapse. Currently, research on the interaction between intestinal helminths and bacteria remains limited, despite its potential contribution to pathological synergy. Drug resistance in conventional STH treatments is a growing concern, highlighting the need for new therapeutic approaches. This study aimed to evaluate the impact of combining the anthelmintic albendazole with the antibiotics piperacillin sodium plus tazobactam on the inflammatory process during chronic experimental trichuriasis. Swiss Webster mice were infected with 150 embryonated *T. muris* eggs. After 35 days, the mice were divided into four groups: Group 1 (antibiotic treatment), Group 2 (anthelmintic treatment), Group 3 (combined treatment), and Group 4 (control, no treatment). After treatments, the mice were euthanized, and different analyses were conducted. Results showed that untreated mice had a significantly higher number of peritoneal macrophages compared to those that received treatment. Antibiotic-treated mice did not show invading bacteria in the epithelial submucosa, unlike untreated infected mice. The groups that received anthelmintic treatment exhibited a higher number of dead worms compared to the antibiotic-only group. Additionally, the combination of anthelmintic and antibiotic treatments demonstrated more effective control of nematode colonization and bacterial translocation, potentially reducing the secondary impacts of the infection, such as bacterial translocation and the associated inflammatory processes. These findings suggest that our results could pave the way for the development of new treatment protocols for STH, integrating both anthelmintic and antibiotic therapies.

## Graphical abstract

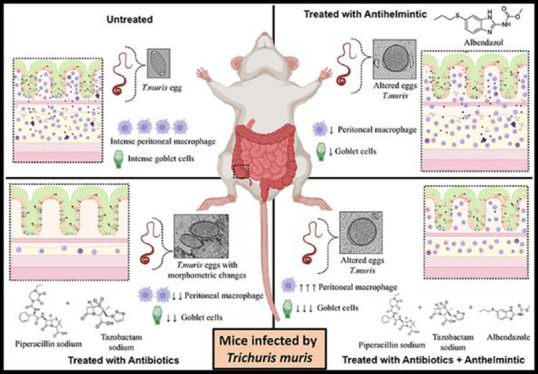

Soil-transmitted helminth infections are among the most common infections worldwide, with over 1.5 billion people infected. They are widely distributed in tropical and subtropical areas where sanitation is poor, with the highest numbers occurring in sub-Saharan Africa, the Americas, China, and East Asia.^1^ More than 267 million preschool-aged children and over 654 million school-aged children live in areas with intensive transmission, requiring both treatment and preventive interventions.^1^

Nematodes of the genus *Trichuris* are highly host-specific, establishing an intratissue niche in the intestines of infected hosts. The role of *Trichuris* species infecting nonhuman primates has been debated, particularly regarding its epidemiological implications. Yao et al.^2^ demonstrated that the African green monkey (*Chlorocebus aethiops sabaeus*) on the Caribbean Island of St. Kitts may serve as a potential reservoir or become infected by another *Trichuris* species. Similarly, Venkatesan et al.^3^ identified *Trichuris incognita*, a species distinct from *Trichuris trichiura* infecting both humans in Côte d’Ivoire and nonhuman primates, highlighting its zoonotic potential. These findings emphasize the importance for monitoring of *Trichuris* infections across species, particularly in countries like Brazil, where sanitation challenges and a diverse range of nonhuman mammals and primates could influence transmission dynamics.^2^

*Trichuris muris* is a parasitic nematode that inhabits the cecum and colon of rodents, exhibiting a direct (monoxenous) life cycle. It serves as an important model for studying host-parasite interactions, particularly in relation to inflammatory responses and immunomodulation, due to the similarities in its pathophysiological effects with those observed in human and veterinary infections.^4^ *T. muris* anchors its anterior end into the intestinal epithelium, causing mucosal ruptures and the formation of syncytial tunnels. This mechanism not only facilitates bacterial translocation into the submucosa but also highlights a complex interplay between the nematode infection and the presence of invasive bacteria.^5,6^ The resulting lesions create pathways for bacterial invasion and contribute to disruptions in the gut microbiota, leading to dysbiosis, which can exacerbate disease severity and further modulate host immune.^7^

These inflammatory processes induce significant tissue alterations, including hypertrophy of the intestinal layers, hematological changes such as decreased hemoglobin levels,^8^ plasma iron deficiency,^9^ and reduced vitamin A synthesis.^10^ Our group has also observed in infected animals a decrease in glucose, iron-deficiency anemia, and leukocytosis.^6^ Additionally, *Trichuris* infection increases muscular contractility of the intestine, with hypercontractility serving as an important innate mechanism for parasite expulsion in resistant mice.^11^

The immune response to *T. muris* infection is critical in determining host susceptibility or resistance during the early stages of infection. In murine models, a protective Th2 response, characterized by cytokines such as IL-4, IL-5, and IL-13, facilitates parasite expulsion. In contrast, a Th1 response, characterized by IFN-γ production, is associated with chronic infection and parasite persistence.^11,12^ In the chronic phase of *T. muris* infection, with a high parasitic load, the immune response becomes more complex, exhibiting a mixed Th1/Th2/Th17 profile. Elevated IFN-γ levels in the cecum indicate that the parasite is capable of modulating the local immune environment, which may contribute to its survival and persistence.^6,7^

In genetically predisposed C57BL/6 APC^min/+^ mice, chronic infection with *T. muris* induces neoplastic alterations, such as increased cell proliferation and carcinogenic cell growth, suggesting a potential role for the parasite as a cofactor in tumor development.^13,14^ This highlights the complex relationship between parasitic infections and host susceptibility to diseases beyond the immediate immune response.^15^

Recent studies have underscored the significant impact of helminth infections, particularly *Trichuris* species, on host immune responses. These infections often lead to immune modulation through excretory-secretory (ES) products, which possess antimicrobial properties that influence the microbial composition in the host’s gut. Although dysbiosis may occur, the primary concern is bacterial translocation, whereby bacteria move from the intestinal lumen into the submucosa or bloodstream. This translocation can exacerbate systemic inflammation and contribute to the development of chronic inflammatory conditions.^7,16^

The main intervention available for controlling soil-transmitted helminth infections (STH) is the periodic administration of anthelmintic drugs, such as mebendazole (MEB), albendazole (ALB), levamisole (LEV), or pyrantel (PYR), recommended by the World Health Organization (WHO). Among these, the benzimidazoles, particularly MEB and ALB, are the most commonly used for treating *Trichuris* infections. While mass drug administration (MDA) programs have been successful in many regions, their effectiveness is limited, especially against *T. trichiura*, with cure rates falling below 90%, which is considered insufficient for eradication. Furthermore, without accurate diagnosis or appropriate dosages, treatment with mebendazole may alter the morphology of *Trichuris* eggs, complicating diagnosis and impeding proper infection control.^17^

Emerging evidence suggests the development of resistance to commonly used anthelmintics in *Trichuris* species. Specific mutations in the β-tubulin gene of *T. trichiura* have been associated with reduced drug susceptibility, particularly to benzimidazoles, highlighting the need for alternative therapeutic strategies.^18^

Albendazole, a carbamate anthelmintic, inhibits tubulin polymerization in parasites, disrupting their microtubule structure, leading to parasite immobilization and death. Although effective for various stages of the parasite lifecycle, it may not completely eliminate the infection in cases of resistance.^19^ As a result, research into new combinations or adjuvant therapies is critical.

In this context, the combination of an anthelmintic with antibiotics, such as piperacillin and tazobactam, has shown promise. Piperacillin, a β-lactam antibiotic, combined with Tazobactam, a β-lactamase inhibitor, extends the antibacterial spectrum and could prove valuable in addressing bacterial translocation during helminth infections. This combination has shown efficacy against both Gram-positive and Gram-negative bacteria, including common enteric pathogens that translocate across the intestinal barrier during dysbiosis.^20,21^ This treatment strategy may help prevent or treat infections caused by bacteria that emerge as opportunistic pathogens during helminth infections, such as *Escherichia coli* , *Klebsiella*, and *Proteus* species.^22,23^

Given these challenges, the present study aims to investigate the combined effect of an anthelmintic (albendazole) and an antibiotic (piperacillin-tazobactam) in the treatment of experimental trichuriasis. The goal is to explore novel therapeutic approaches to improve outcomes by addressing both parasitic infection and associated bacterial translocation. Preliminary findings suggest that this combination therapy significantly reduces parasite burden, improves tissue inflammation, and enhances immune response, presenting a potential strategy to improve therapeutic efficacy in managing trichuriasis.

## Results and Discussion

### Reduction in *Trichuris muris* Egg Shedding following Antibiotic and Anthelmintic Treatment

The quantification of *T. muris* eggs shed in feces revealed a significant percentage reduction in all treated groups compared to the untreated group. The group treated with anthelmintic showed a marked decrease in egg shedding by the seventh day after the treatment. The group receiving antibiotics also demonstrated a considerable reduction in egg numbers, showing a clear difference from the untreated group. Similarly, the group treated with the combination of both drugs exhibited a pronounced decrease in egg shedding, following a pattern comparable to the anthelmintic-only group (Figure 1).

**Figure 1:**
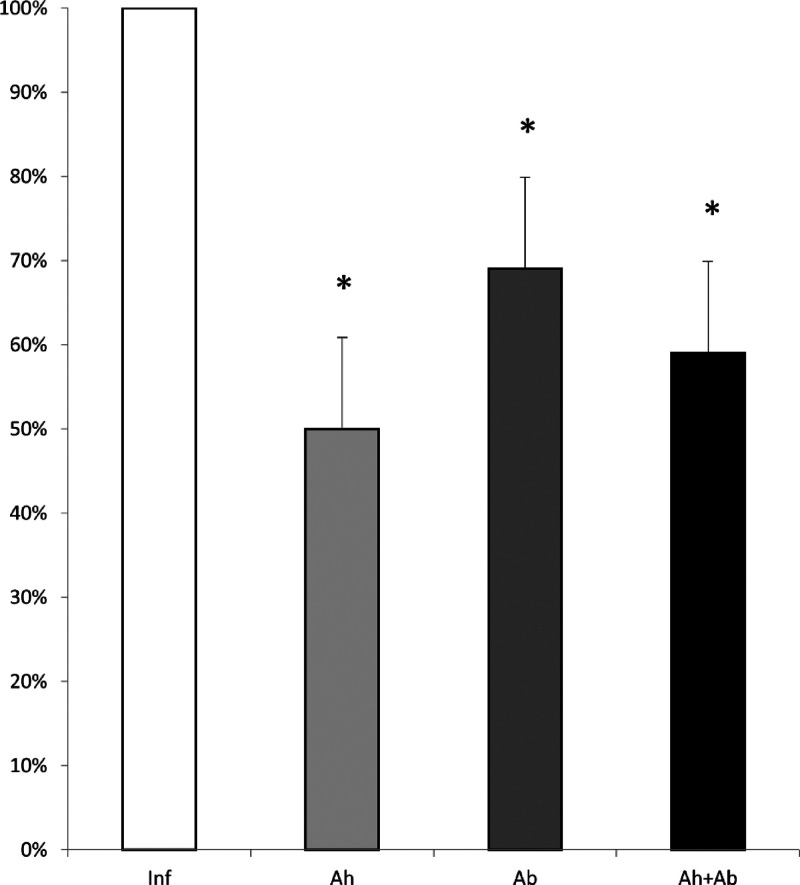
Quantification of the number of *Trichuris muris* eggs eliminated in the feces of treated and untreated mice from the last day of treatment or nontreatment. Inf – infected without treatment; Ah – infected treated with anthelmintic; Ab – infected treated with antibiotic; and Ah + Ab – infected treated with anthelmintic + antibiotic. *Significant difference observed (*P* ≤ 0.05) – *n* = 6. Statistical analysis by *t* test.

### Morphological Alterations in *Trichuris muris* Eggs and Increased Worm Mortality following Anthelmintic Treatment

Regarding parasitic burden, no statistically significant differences were observed in the total worm count between treated and untreated groups (Table 1). However, a significantly higher proportion of dead worms remained attached to the intestinal tissue in animals treated with anthelmintic (*P* = 0.0448) and the combination of drugs (*P* = 0.0374) compared to the untreated and antibiotic-treated groups. These findings are consistent with the significant morphological alterations observed in *Trichuris muris* eggs during stool examinations following anthelmintic treatment. Morphological and morphometric changes were detected as early as the third day of treatment, with the anthelmintic-treated group exhibiting the highest number of altered eggs (Figure 2a–c). In contrast, the antibiotic-treated group exhibited only morphometric changes without any morphological alterations (Figure 2d). The group that received the combined treatment showed alterations in the eggs; however, no significant differences were recorded.

**Table 1:** Quantification of Worms Recovered at Necropsy^a^

groups	live (mean ± S.D.)	dead (%)	total recovered (mean ± S.D.)
Inf	76.8 ± 34.4	11.5	86.8 ± 37.9
Ah	80.8 ± 21.7	19.7^b^	100.6 ± 24.5
Ab	115 ± 20.3	11.4	129.8 ± 21.5
Ah + Ab	74.6 ± 28.8	15.8^b^	88.6 ± 34.7

a Inf – infected without treatment; Ah – infected treated with anthelmintic; Ab – infected treated with antibiotic; Ah + Ab – infected treated with anthelmintic + antibiotic; and SD – standard deviation.

b Significant difference observed (*P* ≤ 0.05) – *n* = 6. Statistical analysis by *t* test.

**Figure 2:**
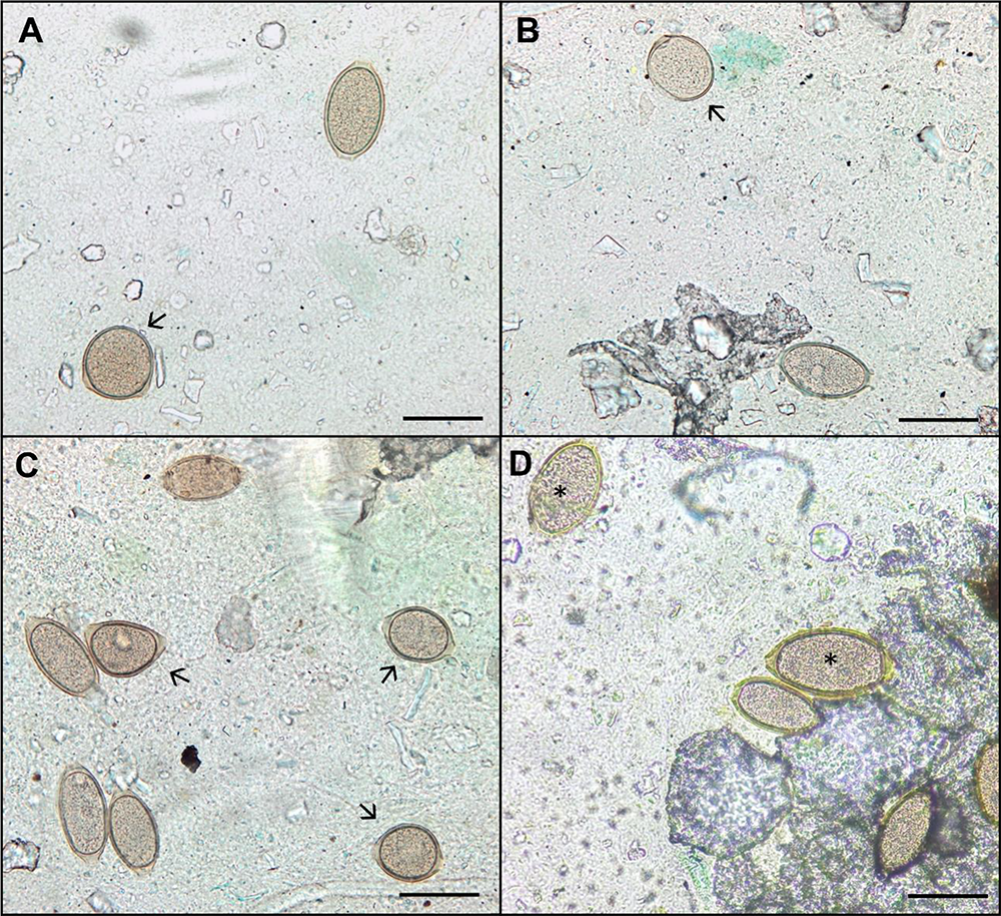
Bright-field light microscopy showing Kato–Katz results with morphologically altered *T. muris* eggs. (a–c) Eggs from animals treated with anthelmintic; (d) eggs from animals treated with antibiotic; arrows – eggs with morphological alterations; asterisk – eggs with morphometric alterations; and scale bar: 50 μm.

An important consideration is that, even after death, *Trichuris* nematodes remain embedded in the intestinal tissue. This contrasts with other helminths that either reside freely in the intestinal lumen or are attached only by their buccal capsule. The tissue attachment of *Trichuris* spp. facilitates continued access to luminal bacteria, sustaining bacterial translocation even after anthelmintic treatment. This persistence of bacterial translocation post-treatment highlights a unique aspect of this infection and underscores the need to explore new treatment strategies, such as combining anthelmintics with antibiotic therapy.

Based on these results, we suggest that the Albendazole induces morphological damage to *Trichuris muris* eggs, interfering with the elimination of viable eggs in the feces and potentially disrupting the parasite’s life cycle. Despite these effects, a notable number of viable adult worms were recovered during necropsy, indicating a low efficiency to the treatment, underscoring the necessity for alternative therapeutic approaches or drug coadministration strategies. Benzimidazole-based anthelmintics, such as Mebendazole and Albendazole, are known to exhibit substantial efficacy against soil-transmitted helminths such as *Ascaris lumbricoides* and hookworms. However, their efficacy against *Trichuris trichiura* remains moderate to low.^24^ Furthermore, the risk of drug resistance must be carefully considered. Widespread and recurrent administration of Albendazole as part of large-scale deworming programs may exert selective pressures favoring the emergence of resistant strains, ultimately diminishing the long-term efficacy of these interventions.^25^ Thus, while the present findings demonstrate the impact of Albendazole on *Trichuris muris*, they also highlight the critical need for exploring alternative or combination therapies to improve treatment outcomes and mitigate resistance development.

### Reduction of Eosinophils in the Blood Smear of Treated Animals

Infected animals submitted to treatment showed a reduction in eosinophil count when compared to the untreated group, but no changes were observed in other cell types (Table 2). A reduction in eosinophils following Albendazole treatment for helminth infections has been observed in several studies.^26–28^ In our blood smear analyses, we observed eosinophilia in infected animals compared to the control group, consistent with the typical immune response to helminth infections. However, the reduction in eosinophil levels in mice treated with antibiotics, despite the antibiotic not directly targeting nematodes, can be explained by the complex interplay between the parasite, bacterial translocation, and the host immune response. *Trichuris* infection promotes bacterial translocation from the intestinal lumen into the tissue due to its attachment into the mucosa, which triggers a robust inflammatory response, including eosinophilia.^6,7^

**Table 2:** Mean Number of Cells Found in Blood Smear Readings^a^

groups	eosinophil (mean ± S.D.)
Inf	6.5 ± 1.6
Ah	3.2 ± 0.8^b^
Ab	4.5 ± 1.4^b^
Ah + Ab	4.5 ± 1.2^b^
*P* value	0.0134^b^

a Inf – infected without treatment; Ah – infected treated with anthelmintic; Ab – infected treated with antibiotic; and Ah + Ab – infected treated with anthelmintic + antibiotic.

b Significant difference observed (*P* ≤ 0.05) – *n* = 6. Statistical analysis by one-way ANOVA.

When antibiotics are administered, they reduce the bacterial load and translocation, thereby diminishing the inflammatory stimulus in the intestinal tissue. This reduction in bacterial-driven inflammation could lead to a decrease in eosinophil recruitment, even though the nematodes remain largely unaffected by the antibiotic treatment. Large numbers of eosinophils reside in the lamina propria of the gastrointestinal tract, where they play a critical role in maintaining the intestinal epithelial barrier function, particularly in the face of inflammation-associated epithelial cell damage. Eosinophils are not only involved in responses to helminth infections but have also been implicated in bacterial infections and other inflammatory conditions.^29^ This suggests that the observed eosinophilia in *Trichuris* infection is not solely a response to the parasite but is also influenced by the secondary bacterial component. Additionally, antibiotic treatment controlled intestinal bacterial colonization and may have eliminated or reduced bacterial translocation, leading to a decrease in eosinophil levels in the bloodstream compared to infected untreated mice. This suggests that eosinophilia could also be associated with bacterial translocation, and the reduction in eosinophils following anthelmintic treatment may be indirectly related to the reduction in worm burden and the Th2-type immune response.^30^

### Treatment Reduces Submucosal Thickening in Infected Animals

The morphometric analysis of the large intestine (cecum) (Figure 3) in all groups revealed that the treatments led to a reduction in submucosal thickening compared to untreated animals, as expected. However, an unexpected finding was a significant reduction in epithelial mucosa thickness, which was observed in the group that received combined treatment (*P* < 0.0001). The submucosa in the infected groups exhibited intense polymorphonuclear and lymphoplasmacytic infiltration, and all treatments resulted in a reduction in submucosal thickness (Figures 3 and 4). In the mucosal layer of the infected mice, an increase in the number and volume of goblet cells compared with the number and volume of goblet cells in the controls was observed (Figure 5).

**Figure 3:**
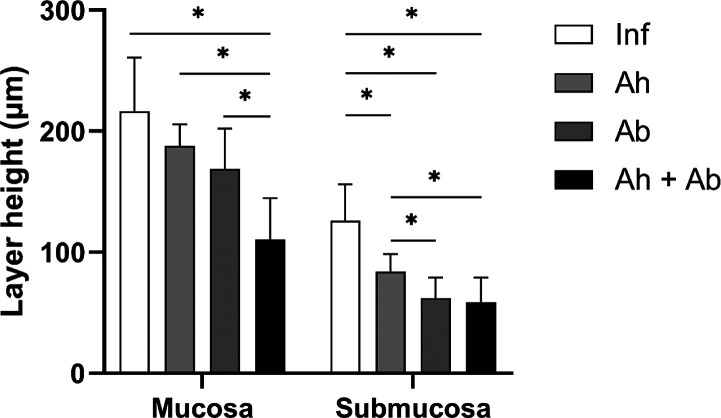
Morphometric analysis of the layers of the large intestine in *T. muris*-infected animals with or without treatments. Inf – infected without treatment; Ah – infected treated with anthelmintic; Ab – infected treated with antibiotic; Ah + Ab – infected treated with anthelmintic + antibiotic; and *significant difference observed (*P* ≤ 0.05) – *n* = 6. Statistical analysis by one-way ANOVA.

**Figure 4:**
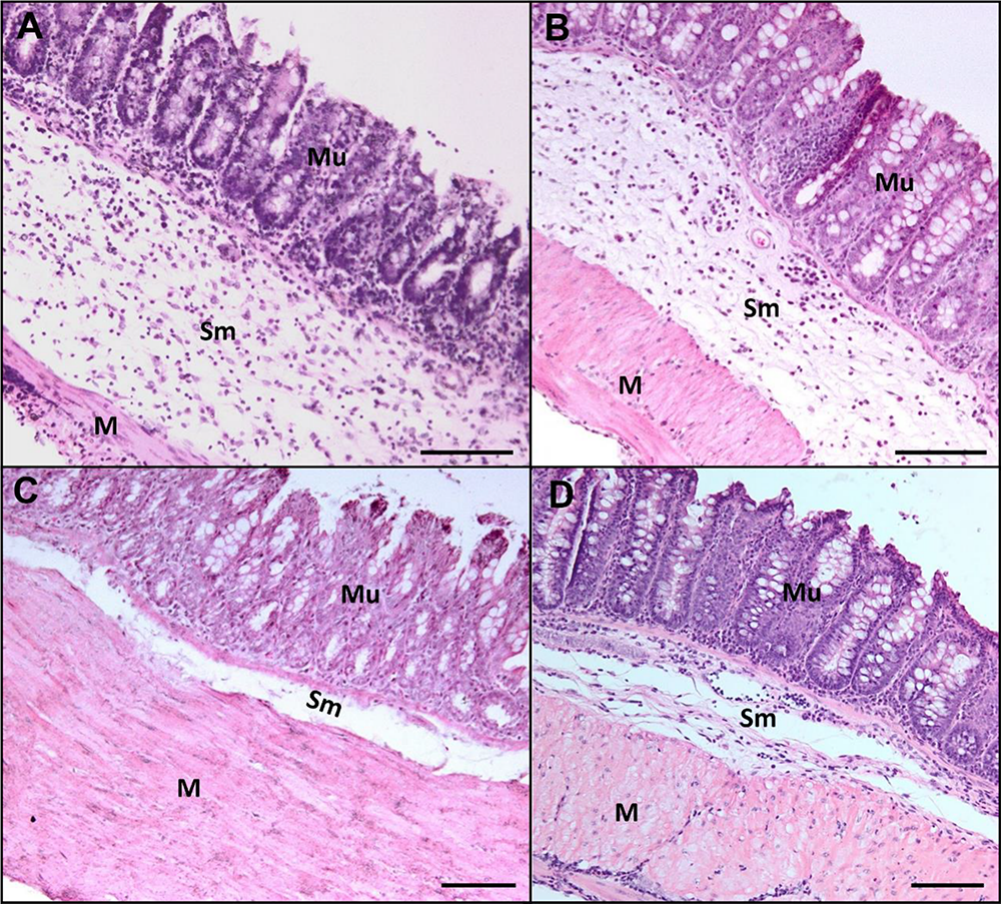
Bright-field microscopy images showing H&E-stained histological sections of the cecum from infected mice. (a) No treatment; (b) treated with anthelmintic; (c) treated with antibiotic; and (d) treated with anthelmintic + antibiotic. Mu – mucosa; Sm – submucosa; and M – muscularis. Scale bar: 100 μm.

**Figure 5:**
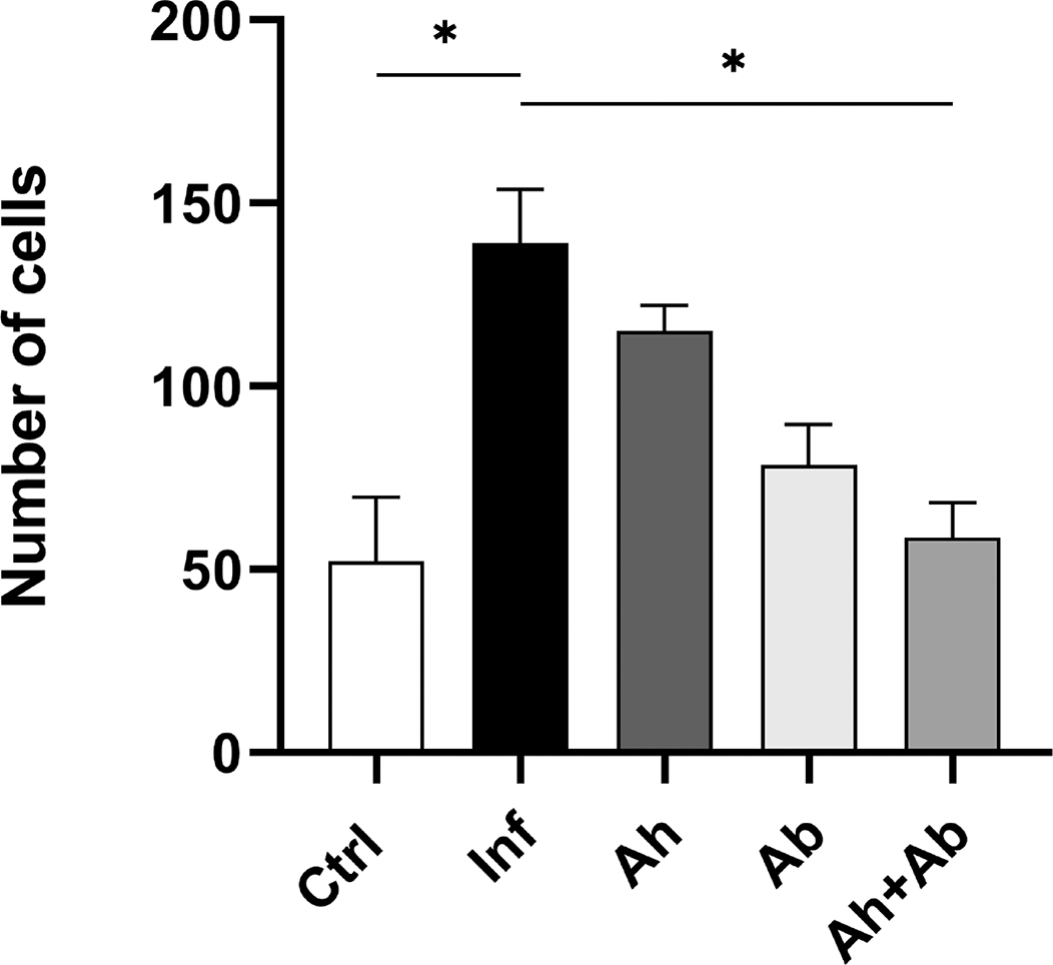
Graph showing goblet cell quantification in the mucosa. Ctrl – control without treatment; Inf – infected without treatment; Ah – infected treated with anthelmintic; Ab – infected treated with antibiotic; and Ah + Ab – infected treated with anthelmintic + antibiotic. *Significant difference observed (*P* ≤ 0.05) – *n* = 6. Statistical analysis performed using one-way ANOVA.

The inflammatory infiltrate results from the nematode’s antigenicity and the tissue damage it causes, combined with bacterial translocation into the intestinal submucosa. This infiltrate is a major factor contributing to the increased thickness of this layer. In untreated infected animals, hyperplasia and hypertrophy of goblet cells were observed in the epithelial mucosa. Our results demonstrated a reduction in epithelial mucosal thickness in the antibiotic-treated groups, highlighting that opportunistic bacteria invading the submucosa are directly linked to the heightened inflammatory response in host tissues during this infection. Additionally, hypertrophy was observed in the muscularis layer, characterized by an increase in muscle fiber size due to stimulated and intensified muscle activity as the host attempts to expel the helminths.^15^

### Treatment Reduces Immune Cell Infiltration

In the morphological analysis of the intestinal submucosa of infected mice, the increased thickening observed through morphometry was attributed to intense inflammatory infiltration, characterized by a significant increase in neutrophils, eosinophils, macrophages, and lymphocytes compared to the control groups. Among the treated groups, significant differences were observed in neutrophils and lymphocytes when compared to the untreated group. Notably, antibiotic therapy reduced lymphocytes compared to the anthelmintic treatment and the combined treatment. The specific impact of the antibiotic was particularly evident in the reduction of eosinophils and macrophages. Interestingly, only when the antibiotic was included in the treatment were these cells significantly reduced; in contrast, no difference was observed in eosinophils and macrophages when comparing the group treated only with anthelmintic to untreated animals. These findings highlight the differential effects of the treatments on inflammatory cell populations, with the antibiotic playing a key role in modulating the immune response (Figure 6).

**Figure 6:**
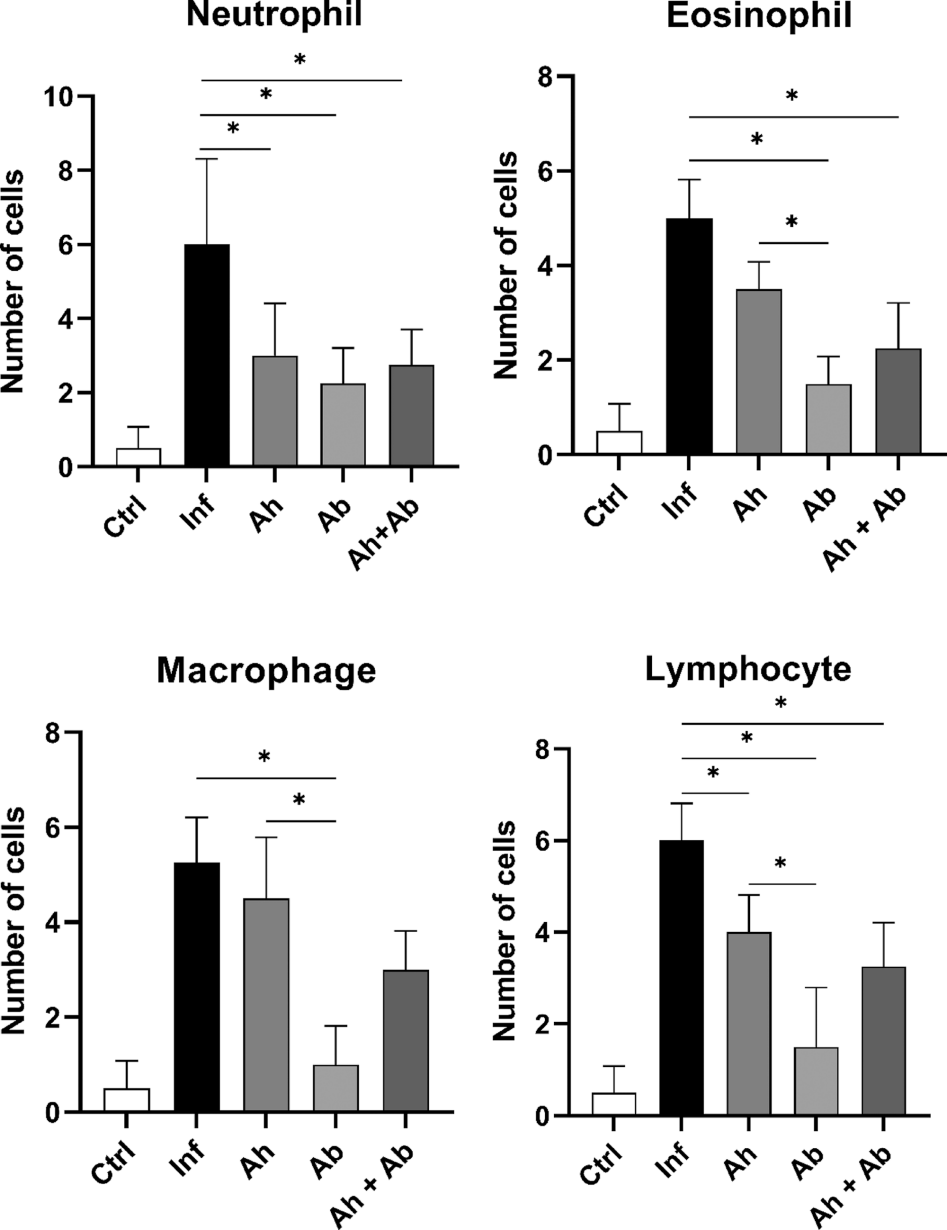
Graph showing the results of identification and quantification of the cells present in the submucosa. Ctrl – control without treatment; Inf – infected without treatment; Ah – infected treated with anthelmintic; Ab – infected treated with antibiotic; and Ah + Ab – infected treated with anthelmintic + antibiotic. *Significant difference observed (*P* ≤ 0.05) – *n* = 6. Statistical analysis by one-way ANOVA.

Morphological characterization of the cells found in the intestinal epithelium showed that the thickness alteration observed in chronically infected mice was promoted by changes in the volume and number of each infected mouse’s own tissue cells and by inflammatory cell infiltration. The histopathological images enabled the identification of a polymorphonuclear cell infiltrate in the intestinal sections of the infected mice, contributing to the increase in the height of the mucosal and submucosal layers.^7^ This polymorphonuclear infiltrate (eosinophils and neutrophils) in the intestinal submucosa, is a finding consistent with previous experimental infections^6^ and cases of human trichuriasis.^31^

The damage caused by the parasite to the intestinal epithelium promotes the release of pro-inflammatory cytokines (such as IL-1β, TNF-α, and IL-6) and chemokines that signal the recruitment of leukocytes.^32,33^ Our results show that treatment with albendazole kills the parasite, thereby interrupting the antigenic stimulation mediated by its excretory/secretory products, reducing tissue damage, promoting the regeneration of the intestinal mucosa, and decreasing bacterial translocation, which consequently leads to a reduction in the recruitment of neutrophils and lymphocytes.^33^

Antibiotic treatment influences the gut microbiota and cytokine levels, thereby impacting the immune response.^34^ Additionally, eosinophils can release extracellular DNA traps that capture and eliminate pathogens.^29,35^ In animals treated with antibiotics, we did not observe bacterial invasion of the tissue, which may explain the reduced eosinophil and macrophage infiltration in the intestinal tissue of these animals.

When an antihelminthic is administered, there may be a decrease in the parasitic load, but this does not eliminate the mediators that promote the activation and survival of eosinophils. Additionally, eosinophils may remain recruited in the tissues as part of a tissue repair process or due to a residual immune response.^35^ Even after the elimination of the parasite, the cytokines and chemokines that attract eosinophils may remain elevated, sustaining their presence.^29^

Another important point is that the population of eosinophils can be regulated by complex interactions with the intestinal microbiota and other components of the immune system, which are not directly altered by antihelminthic medication.

The parasite was observed embedded in the epithelial tunnel of all infected groups, including those treated with anthelmintic, suggesting possible resistance to Albendazole treatment, which may be linked to the intratissue behavior of these nematodes (Figure 7). Notably, a reduction in the submucosal inflammatory infiltrate was observed only in the groups treated with antibiotics, either alone or in combination with anthelmintic. This reduction was not seen in the group treated with anthelmintic alone, highlighting the role of antibiotics in elimination of bacterial translocation, modulating the inflammatory response despite the persistence of the parasite.

**Figure 7:**
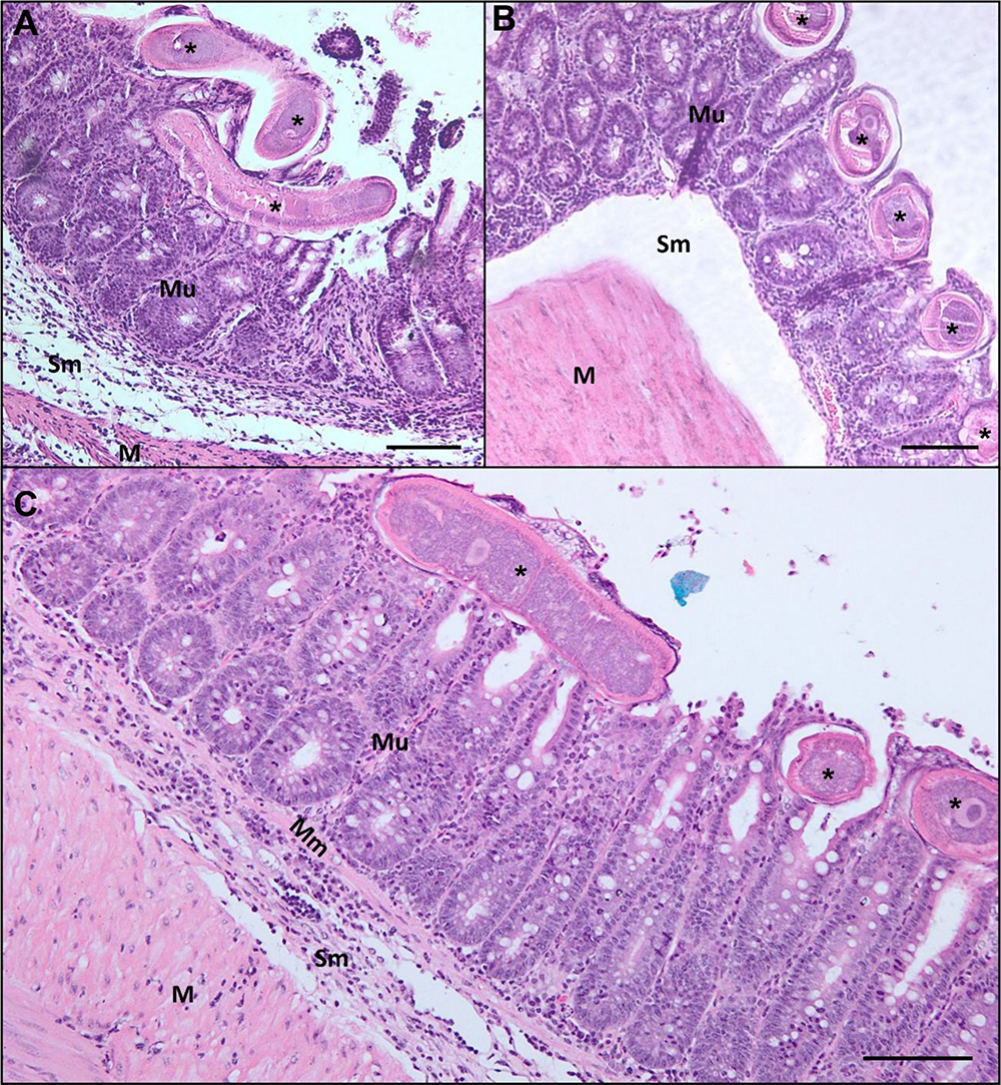
Bright-field microscopy images showing H&E-stained histological sections of the cecum from infected mice with the presence of the parasite in the mucosa. (a) Treated with anthelmintic; (b) treated with antibiotic; and (c) treated with anthelmintic + antibiotic. Arrow – parasite. Scale bar: 100 μm.

Trichuris nematodes exhibit unique characteristics that may contribute to anthelmintic resistance compared to other helminths. Albendazole inhibits β-tubulin polymerization, disrupting microtubule formation and impairing glucose uptake, which leads to ATP depletion and ultimately causes parasite death by starvation.^36,37^ The anterior portion of *Trichuris* spp. is attached in the intestinal mucosa, forming an epithelial tunnel structure, while the posterior portion extends freely into the intestinal lumen.^38^ This strategy suggests that *Trichuris* spp. can shield the anterior region from drugs with poor mucosal absorption, which are typically concentrated in the lumen, while still being exposed to both mucosal and luminal anthelmintic drugs. The bacillary band, located in the anterior region, consists of bacillary glands, cuticular inflations, and stichocytes.^39^ This structure was associated with detoxifying anthelmintic compounds, enabling the parasite to survive conventional doses of benzimidazoles, which are typically effective against other nematodes. This detoxification mechanism may contribute to the development of drug resistance, posing significant challenges for the treatment of trichuriasis.^38,39^

### Reduction in Peritoneal Macrophage Level in Treated Animals

All treated groups receiving anthelmintics, antibiotics, or both showed a significant reduction in peritoneal macrophages compared to the untreated infected group (Figure 8). The reduction of peritoneal macrophages observed in mice infected with *Trichuris muris* is supported by results indicating that all treated groups—those receiving anthelmintics, antibiotics, or both—showed a significant decrease in macrophages compared to the untreated infected group. This challenges the idea that macrophage activation in trichuriasis is primarily driven by bacterial translocation, suggesting instead that both the behavior of the nematode and its soluble antigens play a crucial role in macrophage recruitment. If bacterial translocation were the primary driver of activation, only treatments including antibiotics should have significantly reduced macrophage activation. However, the reduction observed with anthelmintics indicates that nematode-derived factors, such as excretory/secretory (ES) products, are decisive. Thus, anthelmintics may reduce worm burden and ES release, indirectly dampening macrophage activation, while antibiotics likely have a secondary effect by mitigating bacterial costimulation. This mechanism highlights the complexity of the immune response to helminth infections and the importance of considering multiple factors in the dynamics of peritoneal macrophages under infection conditions.^6,40,41^

**Figure 8:**
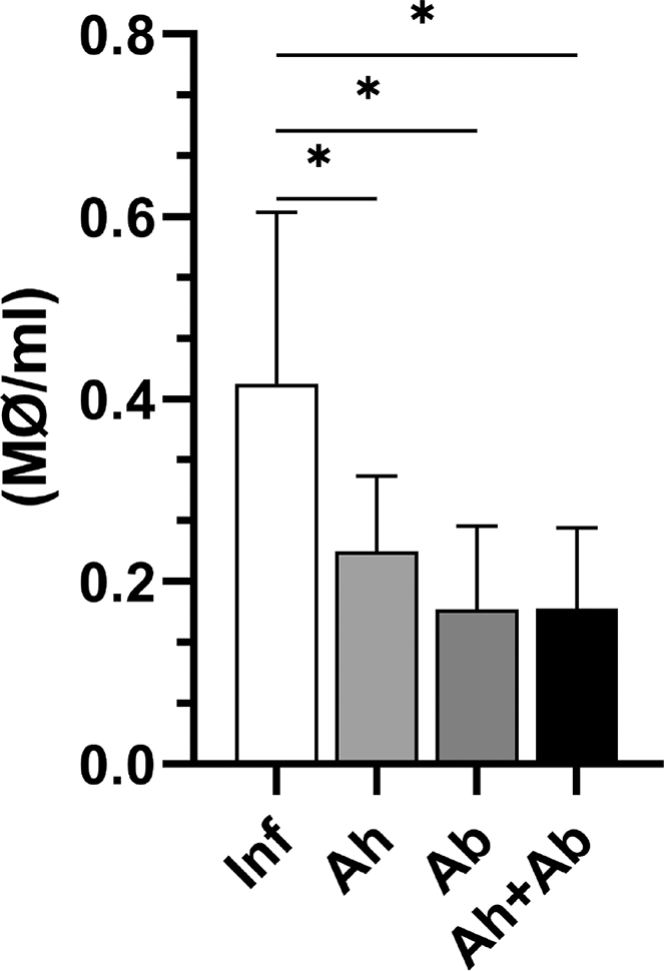
Peritoneal macrophage concentration in infected mice subjected to different treatments or no treatment. Inf – infected without treatment; Ah – infected treated with anthelmintic; Ab – infected treated with antibiotic; and Ah + Ab – infected treated with anthelmintic + antibiotic. *Significant difference observed (*P* ≤ 0.05) – *n* = 6. Statistical analysis by *t* test.

Macrophages are central to the immune response in trichuriasis, particularly in the mucosa and submucosa, where they interact with both bacteria and nematode-derived products. Chronically infected mice exhibit heightened macrophage reactivity, releasing elevated nitric oxide (NO) upon LPS stimulation^6,42^ indicating an activated state likely driven by persistent nematode antigens rather than bacterial translocation alone. The increased recruitment of monocytes and neutrophils further suggests a state of low-grade peritonitis, highlighting the systemic impact of chronic infection.^7^

As the infection progresses, chronic inflammation in the cecum leads to intestinal thickening and the systemic dissemination of nematode antigens, sustaining macrophage activation. Cytokine profiling supports this, showing elevated TNF-α, IL-6, and IL-10 levels during the acute phase, followed by increased NO production in the chronic phase, reflecting a macrophage phenotype that is both antimicrobial and immunomodulatory.^6,7^ These findings align with the idea that *T. muris* employs immunoregulatory strategies to balance inflammation, benefiting both parasite survival and host tissue integrity. The decrease in peritoneal macrophages following treatment with anthelmintics and antibiotics suggests that the nematode might be utilizing strategies to modulate the host immune response. By doing so, *T. muris* can minimize excessive inflammation that might otherwise lead to tissue damage, thereby ensuring its survival within the host. The soluble antigens released by the parasite likely play a role in this regulation, as they can influence the activation and polarization of macrophages, promoting a more tolerogenic environment. This helps maintain a balance between effective immune defense and tissue preservation, illustrating a sophisticated host-parasite interaction where both the parasite and the host may benefit.^42^

Peritoneal macrophage alterations strongly indicate the systemic impact of trichuriasis, underscoring the interplay between the nematode and microbiota in driving inflammation. Previous studies proposed that nematode movement facilitates bacterial translocation.^7^ Our data confirm that *T. muris* significantly influences peritoneal macrophage dynamics. Anthelmintic treatment reduces ES production and nematode motility, thereby limiting bacterial translocation from the intestinal lumen to underlying tissues. Antibiotic treatment alone decreases microbial availability, suggesting that nematode-secreted immunomodulatory substances are sufficient to control minimal bacterial translocation or tissue damage.

## Conclusions

In conclusion, when infected mice received both treatments, the synergistic effects of nematode activity and bacterial translocation were completely controlled. This insight refines our understanding of trichuriasis-associated inflammation and opens new avenues for developing improved treatment strategies for intestinal parasite infections.

## Materials and Methods

### Ethics Statement

The present study was submitted and approved by the Animal Experimentation Ethics Committee of the Roberto Alcantara Gomes Institute of Biology (State University of Rio de Janeiro - UERJ), under protocol number CEUA/021/2022.

### Experimental Infection

The eggs of *Trichuris muris* were incubated at 28 °C for 30 days in sterile water to allow for embryonation and become infective. These eggs were then used to experimentally infect 68 male Swiss Webster mice at 4 weeks of age, with all mice infected with a high parasite load by gavage with 150 embryonated eggs of *Trichuris muris* in 200 μL of sterile water. To establish a parasitic load in chronically infected mice, on days 7, 9, and 11 postinfection, infected and control mice were intramuscularly inoculated with 50 μL of Diprospan (betamethasone dipropionate 5 mg/mL and betamethasone disodium phosphate 2 mg/mL) to induce immunomodulation. This corticosteroid has a biological half-life of 36–54 h and is fully eliminated within approximately 10 days.^43^ Therefore, it does not have pharmacological relevance for the experiments conducted. Between the 30th and 45th day after infection, fecal examinations were performed by spontaneous sedimentation, Hoffman, Pons and Janer or Lutz method to confirm the establishment of chronic phase infection.

### Safety Assessment of Coadministration

To evaluate potential drug–drug interactions arising from the coadministration of Piperacillin + Tazobactam (Figure 9) and Albendazole (Figure 10), an in silico analysis was conducted using the Drugs.com Drug Interactions Checker,^44^ which is frequently used in pharmacological and toxicological studies to evaluate molecular-level interactions based on curated clinical pharmacology data, no clinically significant pharmacokinetic or pharmacodynamic interactions were identified between Piperacillin + Tazobactam and Albendazole. The analysis indicated no risk of increased toxicity, reduced efficacy, or other relevant forms of mutual interference. These findings suggest that the concomitant administration of both drugs is safe within the context of experimental trichuriasis treatment.

**Figure 9:**
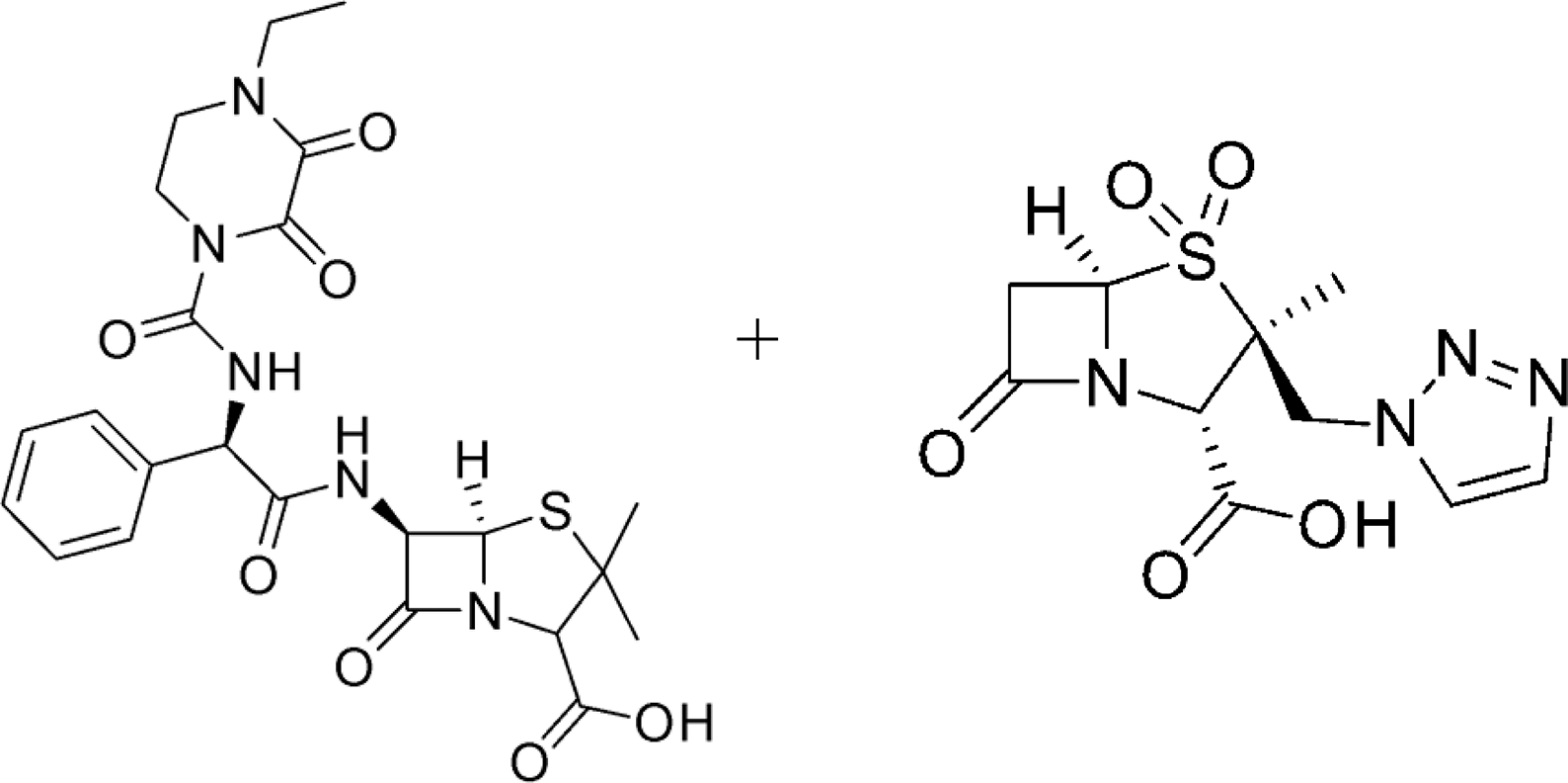
C_23_H_11_N_5_NaO_7_S piperacillin sodium + C_10_H_11_N_4_NaO_5_S tazobactam sodium.

**Figure 10:**
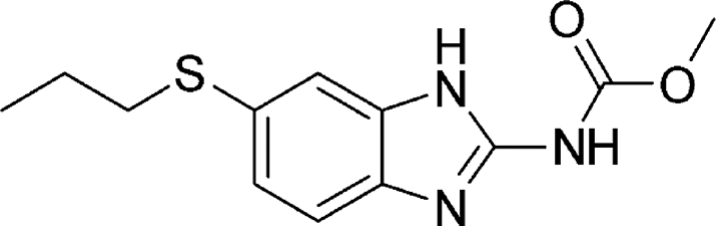
C_12_H_15_N_3_O_2_S albendazole.

### Drugs Administration

After confirmation of the presence of eggs in the feces, the groups were separated again, where some were subjected to treatment with the anthelmintic albendazole at a concentration of 100 mg/kg^45^ and/or the antimicrobial piperacillin sodium + tazobactam sodium at a concentration of 8 mg/mL (Eurofarma),^46^ with their respective controls. The animals that did not receive treatment were inoculated with sterile 0.9% sodium chloride. The treatment with the antimicrobial was for 7 days, and the treatment with the anthelmintic was a single dose, starting on the same day as the first dose of the antimicrobial. With their respective treatments, the groups were separated as follows:

Control without treatment (Ctrl) – 6 animals;
Control treated with anthelmintic (Ctrl Ah) – 6 animals;
Control treated with antibiotic (Ctrl Ab) – 6 animals;
Control treated with anthelmintic and antibiotic (Ctrl Ah + Ab) – 7 animals;
Infected without treatment (Inf) – 10 animals;
Infected treated with anthelmintic (Ah) – 11 animals;
Infected treated with antibiotic (Ab) – 11 animals;
Infected treated with anthelmintic and antibiotic (Ah + Ab) – 11 animals.

### Stool Examination and Study Design

The Kato-Katz^47^ is a quantitative laboratory method adopted by the World Health Organization (WHO) for the diagnosis of various parasitic infections, being a tool of clinical and epidemiological relevance, since it allows the quantification of the number of eggs and, consequently, the estimation of the individual’s parasite load through the calculation of eggs per gram of feces (EPG). The animal feces were collected on two different days:

On the day of treatment (day 0);
On the last day of treatment (day 7);

From the freshly collected feces, a specific Kato-Katz kit was used, where the feces were pressed onto a newspaper with the nylon screen of the kit so that only the eggs and small debris passed through the screen. Then, with the quantifying plate properly positioned on top of a slide, all the material that passed through the screen was transferred to the plate with the aid of a spatula, where these feces completely covered the hole in the plate. Subsequently, the plate was carefully removed from the slide and a cellophane coverslip impregnated with malachite green was used, where this slide was then turned upside down and pressed against the newspaper to fix the coverslip. After preparation, the slides were read under a conventional light microscope (all fields).

### Recovery of Worms and Gut Histology: Morphometric and Morphological Experiments

The cecum was carefully opened, washed in 0.9% saline solution to remove the feces, and then the worms (still attached) were counted as total worms, dead worms, and live worms. Large intestine fragments taken from the cecum region were fixed in 8% formaldehyde at pH 7.4 for 24 h and transferred to 4% formalin. The tissue was dehydrated in a graded ethanol series (30% to absolute), subjected to diaphanization with xylene (Merck), and embedded in paraffin (Sigma-Aldrich). Tissue sections (5 μm) were obtained and stained with hematoxylin-eosin (Sigma-Aldrich), Giemsa (Merck), and periodic acid-Schiff (PAS). Morphometric and morphological analyses were performed using the software Bel View (version 6.2.3.0; Bel Engineering, Monza, Italy), and images were obtained using a Nikon Eclipse 80i microscope. The three cecum layers in five different areas in six animals from each group were measured at random. For morphological experiments in the submucosa, the cells were quantified and identified in randomly fields in six animals. For goblet cell quantification, crypts of Lieberkühn were randomly selected from six animals from each group and analyzed.

### Macrophage Experiments

Peritoneal macrophages were obtained by lavage of the peritoneal cavity with 10 mL of Dulbecco’s modified Eagle medium (DMEM; Gibco, USA). After isolation from the peritoneal cavity, the monocytes-macrophages pass through an adhesion process and need to be maintained in culture for at least 3 days for stabilization. The number of total viable cells of mice, infected with *T. muris* or not, was determined using trypan blue (Sigma-Aldrich) in a Neubauer chamber. The numbers of peritoneal macrophages and cells of the RAW 264.7 macrophage cell line were counted (2 105 cells/well), and the cells were cultured in DMEM supplemented with 10% FBS, 100 U/ml penicillin, 100 g/mL of streptomycin, and 2 mM glutamine in 24-well plates.

### Blood Smear Preparation

Peripheral blood was collected from the tail of the animals, and an aliquot of approximately 5 μL was placed onto a clean, degreased glass slide. The blood was spread at a 45° angle using a spreading slide to ensure that the cells remained within the slide area, facilitating analysis. After drying at room temperature, the cells were fixed and stained using a hematology staining kit, according to the manufacturer’s instructions. The blood smears were analyzed qualitatively under a light microscope (Olympus Standard CX-21) for differential leukocyte counting. The analysis was performed using the modified Ameia method to account for the uneven distribution of leukocytes between the center and edge of the smear, as different types of leukocytes do not distribute uniformly across the slide.

### Statistical Analyses

All uninfected control groups (untreated; treated with anthelmintic; treated with antibiotic; and treated with both anthelmintic and antibiotic) were compared and no statistically significant differences were detected. Consequently, only data from the untreated, uninfected control group are presented in the plotted results. Comparisons among experimental groups were performed using the nonparametric Mann–Whitney test, parametric Student’s *t*-test, and one-way analysis of variance (ANOVA), as appropriate. A *P* value of <0.05 was considered statistically significant. Statistical analyses were conducted using GraphPad Prism software (version 5) and GraphPad InStat software (GraphPad, USA).
